# Supplementary figures and images for: Characterization of the Regulatory Network under Waterlogging Stress in Soybean Roots via Transcriptome Analysis
Source: Plants (Basel). 2024 Sep 10;13(18):2538. doi: 10.3390/plants13182538 (PMC11435190; doi:10.3390/plants13182538)

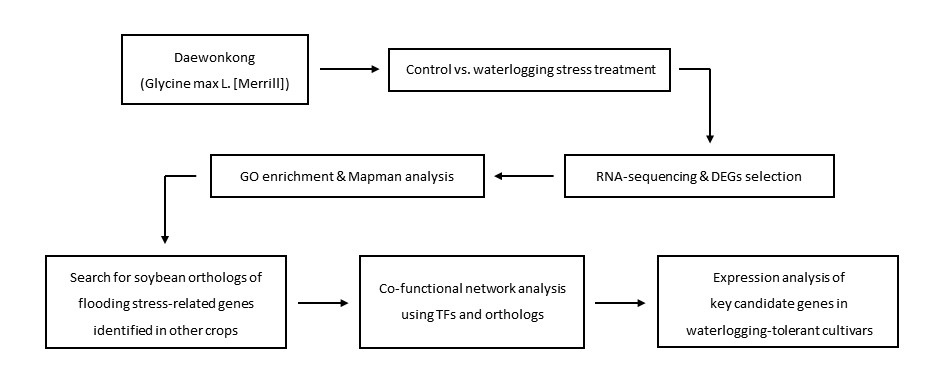

Supplement: Supplementary file 1 [file plants-13-02538-s001.zip › Figure S1_revise.jpg]

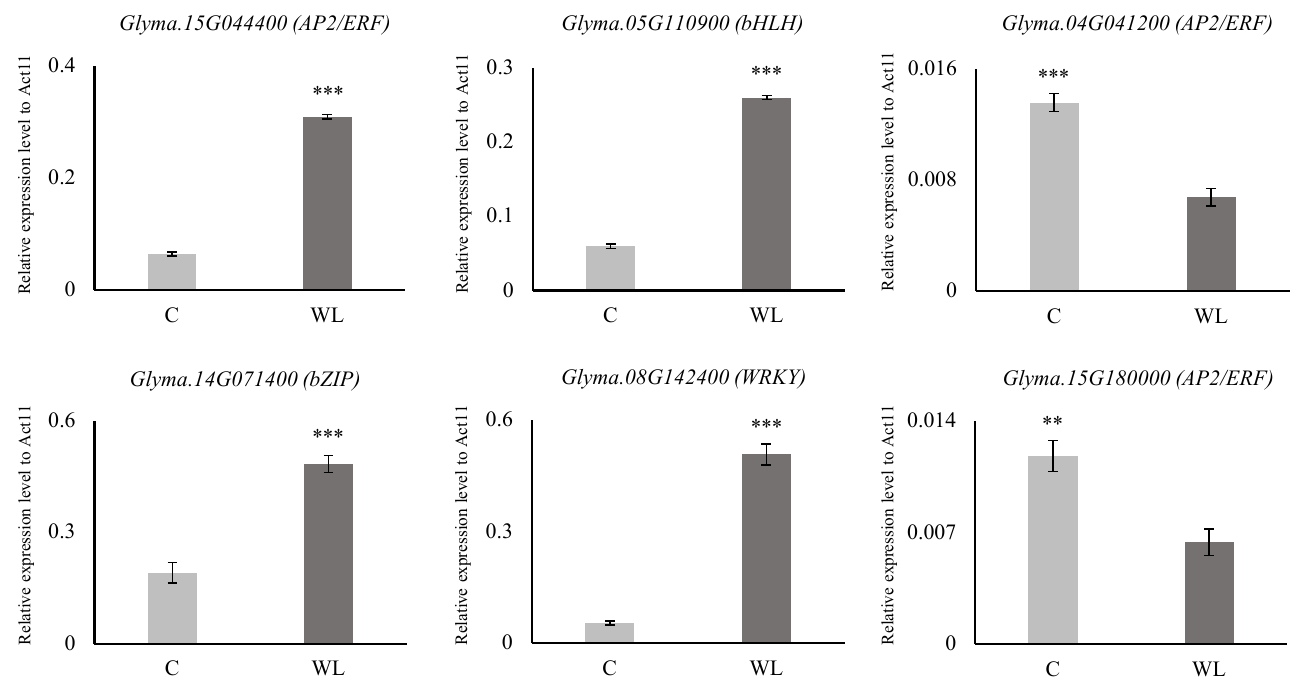

Supplement: Supplementary file 1 [file plants-13-02538-s001.zip › Figure S2_2nd rev.png]

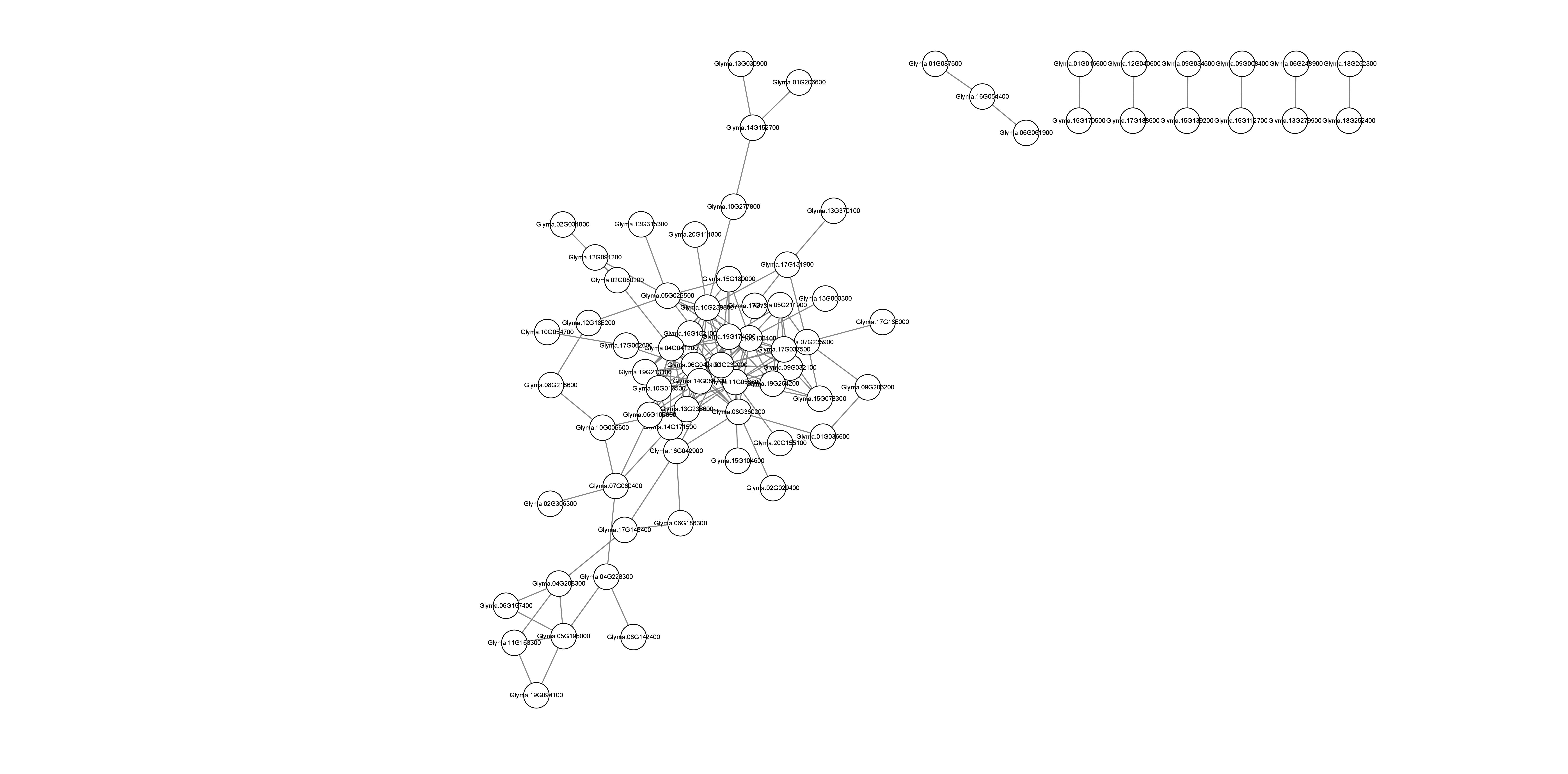

Supplement: Supplementary file 1 [file plants-13-02538-s001.zip › Figure S3_2nd rev.jpeg]
